# Bibliography

**Published:** 1907-02-15

**Authors:** 


### BIBLIOGRAPHY.

Atlas and Text-Book of Dentistry, Including Diseases of the Mouth. By Gustav Preiswerk, M.D., Ph.D., of the University of Basel. Authorized Translation from the German. Edited by George W. Warren, A.M., D.D.S., Professor of Principles and Practice of Operative Dentistry, Pennsylvania College of Dental Surgery. With 103 Colored Figures and Forty-four Plates and 152 Text Illustrations. Philadelphia and London: W. B. Saunders Company. 1906. Cloth, $3.50.

This is one of the series of Hand-Atlases published by the Saunders Co. The book is not a large folio atlas, as one might infer from the title, but is a handy 12 mo. size of 344 pages, besides the many inserts. The author is a medical man and presents the matter almost entirely freed from technical procedures, and since it presents the subject from the German standpoint, it is very interesting and instructive to the American reader. It will probably find little use as a text-book in our educational institutions, but it ought to be in the library of every practitioner. The American editor has added many things to make the book accord with our ideas, but we are glad that he has had the good judgment to give it to us largely in the original form. There are some, perhaps many, things that we cannot accept, but there is so much that is true and suggestive that it is a most valuable contribution to our literature. The book is profusely illustrated with artistic lithographic illustrations, in fact we have never seen a dental book more artistically illustrated. One illustration, a longitudinal section of the pulp, showing in colors the plexus of blood vessels, nerves and cells is well worth the price of the book—in fact all the colored illustrations are remarkably well done, and we are surprised that it can be published for the price asked. The few references to technical methods are not such as would be approved here, and we were at first inclined to censure the American editor for allowing them to stand, but as we have already said, we think they will only lend interest to the reading of the work by the American practitioner. The plan of the work is to present first the Anatomy, Histology and Physiology of the teeth and associated parts; then the diseases are discussed and illustrated with many drawings and colored plates, in fact fully three-fourths of the text is devoted to this consideration. The technical matter occupies about forty pages, except where it has to be considered in connection with caries and pulp treatment. It is really a most interesting work, and we sincerely trust it may have a large sale as it will be the means of awakening new interest in the whole subject of dental pathology.

